# Association of General Anesthesia Exposure with Cognitive Outcomes in Older Adults: A Cross-Sectional Study in a Korean Cohort

**DOI:** 10.3390/jcm15155864

**Published:** 2026-07-27

**Authors:** Kayoung Song, Min-Seung Park, Seong Yoon Kim

**Affiliations:** 1 Department of Psychiatry, Veteran Health Service Medical Center, Seoul 05368, Republic of Korea; 2 Department of Laboratory Medicine, Kangbuk Samsung Hospital, Sungkyunkwan University School of Medicine, Seoul 03181, Republic of Korea; 3 Department of Psychiatry, Asan Medical Center, University of Ulsan College of Medicine, Seoul 05505, Republic of Korea

**Keywords:** general anesthesia, cognitive decline, dementia, clinical dementia rating, global deterioration scale

## Abstract

**Background**: The relationship between general anesthesia (GA) exposure and cognitive function remains controversial. This study aimed to investigate the association between GA exposure and cognitive severity, as measured using the Clinical Dementia Rating Sum of Boxes (CDR-SB) and Global Deterioration Scale (GDS), across different diagnostic groups. **Methods**: We used a de-identified dataset from the Korea Dementia Research Center’s Trial Ready Registry (KDRC TRR). Participants were classified as having normal cognition, mild cognitive impairment (MCI), or dementia. Logistic regression analyses were performed to identify the variables associated with CDR-SB and GDS scores within each group. Linear regression analyses were also performed for the amyloid positron emission tomography standardized uptake value ratios (SUVRs). **Results**: Among 688 participants, 258 were classified as having normal cognition, 245 as MCI, and 185 as dementia. In the normal group, GA exposure was associated with higher CDR-SB (OR = 2.33 [95% CI: 1.06–5.08, *p* = 0.032]) and GDS scores (OR = 2.29 [95% CI: 1.16–4.55, *p* = 0.017]). In addition, in this group, GA exposure was significantly associated with higher SUVRs in multivariable analyses (β = 0.09, 95% CI: 0.004–0.18, *p* = 0.040). No consistent associations were observed in the MCI or dementia groups. **Conclusions**: GA exposure after the age of 50 was associated with greater cognitive severity and higher amyloid burden in cognitively normal individuals. Prospective longitudinal studies incorporating detailed anesthetic, surgical, and perioperative data are required to clarify the nature of this association.

## 1. Introduction

As the world population ages, cognitive decline has become an important healthcare issue. Postoperative cognitive decline is common among older adults and ranges from 25% to 70% in the first week after surgery under general anesthesia (GA) [1,2]. For a substantial proportion of patients, cognitive changes are not merely transient; approximately 10–40% of elderly individuals continue to experience persistent or long-lasting cognitive decline three months after surgery [1,2]. These observations raise concerns about the potential long-term cognitive consequences of GA exposure in aging populations.

Preclinical animal studies have suggested plausible biological mechanisms underlying the association between GA exposure and cognitive dysfunction and Alzheimer’s dementia-related neuropathological changes [1,3,4,5,6]. However, clinical evidence in humans remains inconsistent [7,8,9,10,11]. Epidemiological data including large-scale cohort studies from South Korea and China support a significant link between GA exposure and increased risk of long-term cognitive impairment, dementia, and structural brain alterations [7,8,12,13]. In contrast, a recent large meta-analysis challenged this assumption and reported no significant association between GA exposure and subsequent cognitive decline or dementia in diverse global populations [11].

Despite growing interest in the association between GA exposure and cognitive outcomes, critical knowledge gaps remain. Most previous studies have been limited by small cohorts, heterogeneous outcome measures, lack of expert clinical cognitive assessment, omission of key factors such as APOE ε4 carrier status and amyloid biomarkers, and/or insufficient stratification by cognitive stage.

Accordingly, the present study used data from the Korea Dementia Research Center’s Trial Ready Registry to examine the association between GA exposure after the age of 50 and cognitive severity among individuals with normal cognition, mild cognitive impairment (MCI), and dementia. By stratifying the participants according to their cognitive stage and adjusting for demographic, genetic, and clinical covariates, we aimed to provide a more reliable estimate of this association.

## 2. Materials and Methods

### 2.1. Data Source and Participants

The data for this study were obtained from the Korea Dementia Research Center’s Trial Ready Registry (KDRC TRR), which is supported by the Ministry of Health and Welfare and the Ministry of Science and ICT, Republic of Korea [14]. The registry provides comprehensive anonymized clinical and demographic characteristics, diagnostic classifications, medical histories, clinical assessments, laboratory results, and neuroimaging findings. MCI and dementia were diagnosed in accordance with the National Institute on Aging–Alzheimer’s Association (NIA-AA) criteria [14,15,16].

The following exclusion criteria were applied to this platform: individuals with psychiatric disorders; major neurological or severe systemic illnesses potentially affecting cognitive function; and/or severe visual or hearing impairments that could interfere with neuropsychological evaluation were excluded. Participants who could not undergo brain Magnetic Resonance Imaging (MRI) or Positron Emission Tomography (PET) imaging were also excluded. In addition, individuals with secondary causes of cognitive impairment were identified via laboratory tests such as vitamin B12 deficiency, positive syphilis serology, and abnormal thyroid, renal, or hepatic functions. Patients with structural brain abnormalities evident on MRI including territorial infarctions, multiple lacunar infarcts, intracranial hemorrhages, more than five cerebral microbleeds, large hemorrhages (≥10 mm), brain tumors, hydrocephalus, or severe white matter hyperintensities (assessed using the modified Fazekas scale), were also excluded [14].

**Figure 1:**
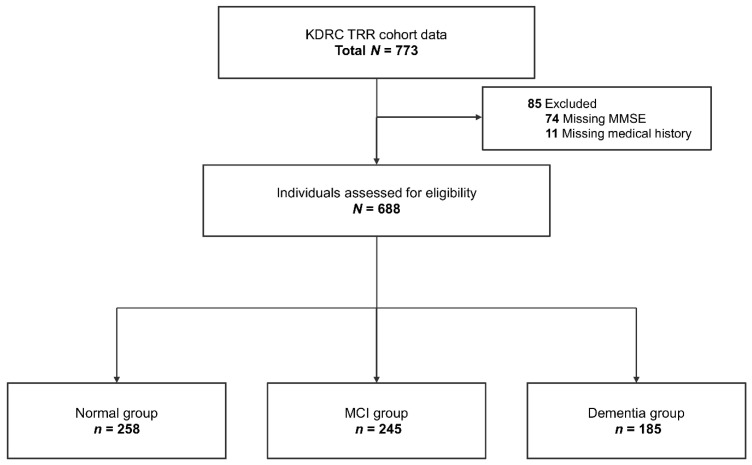
Schematic flow of the study.

A total of 773 individuals were initially retrieved from the de-identified KDRC TRR dataset. Participants with missing Mini-Mental State Examination (MMSE) scores (*n* = 74) or incomplete medical histories (*n* = 11) were excluded. The remaining 688 individuals with complete data were classified into three diagnostic groups (normal, MCI, and dementia). A schematic of participant selection and group allocation is shown in Figure 1.

All experimental protocols were approved by the Institutional Review Board of the Veterans Health Service Medical Center, Seoul, Republic of Korea (Approval No. 2024-02-019, approval date: 11 March 2024), in accordance with the relevant guidelines and regulations. This study complied with the principles of the Declaration of Helsinki. Initial IRB approval waived the requirement for informed consent owing to the retrospective nature of the study.

### 2.2. Variables for Analysis

The following variables were extracted for analysis: age, sex, years of education, family history of dementia, GA exposure after the age of 50 years, diabetes mellitus, hypertension, dyslipidemia, ever smoking, history of alcohol consumption, comorbidity count, MMSE, Clinical Dementia Rating Sum of Boxes (CDR-SB), Global Deterioration Scale (GDS), APOE ε4 carrier status, and amyloid PET findings including amyloid positivity and standard uptake value ratios (SUVRs). Comorbidity count was calculated as the unweighted sum of major medical comorbidities including heart disease, cerebrovascular disease, pulmonary disease, liver disease, and malignancy. Amyloid PET positivity was determined by visual assessment. Amyloid burden was quantified using SUVRs, with higher values indicating greater cerebral amyloid burden.

### 2.3. Primary Outcomes: Clinical Dementia Rating Sum of Boxes (CDR-SB) and Global Deterioration Scale (GDS)

The CDR is a global scale used to assess cognitive changes and to evaluate the presence and severity of dementia. It comprises multiple domains, each rated separately, and classifies dementia severity as very mild (CDR 0.5), mild (CDR 1), moderate (CDR 2), or severe (CDR 3) [17]. The scores from each domain can be summed to yield the CDR-SB, which provides a more granular measure of cognitive impairment than the global CDR score alone [17]. The CDR-SB score ranges from 0 to 18 points, increases with dementia severity, and is widely used as a measure of dementia severity in clinical and research settings [17,18]. The GDS is a seven-stage scale that measures the progression and stage of primary degenerative dementia [19]. The GDS comprises seven stages ranging from stage 1 (no cognitive decline) to stage 7 (very severe cognitive decline) [19].

### 2.4. Exposure Variable

GA exposure was defined as a self-reported history of GA after the age of 50 years and was analyzed as a binary variable (yes/no).

### 2.5. Statistical Analysis

Group comparisons across diagnostic groups were performed using the Kruskal–Wallis tests for continuous variables (age, years of education, comorbidity count, MMSE, CDR-SB, and GDS scores, and SUVRs) and the chi-square tests for categorical variables (sex, family history of dementia, GA exposure, smoking and alcohol history, diabetes mellitus, hypertension, dyslipidemia, APOE ε4 carrier status, and amyloid PET positivity).

CDR-SB and GDS are bounded ordinal measures with floor and ceiling effects, respectively. To accommodate these characteristics, participants within each diagnostic group were classified into low and high CDR-SB and GDS categories using severity thresholds derived from previous literature [19,20,21,22]. The group-specific cutoff values and the corresponding distributions by GA exposure are summarized in Table 1.

Nine participants with missing GDS scores were excluded from GDS-related analyses. Sensitivity analyses were performed using a three-category classification (low, intermediate, and high) (Table S1).

Univariate and multivariable logistic regression analyses were performed using categorized CDR-SB and GDS scores as dependent variables. Amyloid PET SUVR was analyzed using linear regression. Within each diagnostic group, the unadjusted CDR-SB and GDS scores were compared between GA-exposed and non-exposed participants using the Wilcoxon rank-sum test.

All statistical analyses and data visualizations were performed using R software (version 4.5.0). Odds ratios (ORs) and regression coefficients (β) with corresponding 95% confidence intervals (CIs) were reported. Statistical significance was set at *p* < 0.05.

## 3. Results

### 3.1. Comparison of Participant Characteristics Across Diagnostic Groups

A total of 688 participants were enrolled in the study, of whom 433 (62.9%) were female with a median age of 74 years (Table 2). The participants were classified into three groups: normal cognition (*n* = 258, 37.5%), MCI (*n* = 245, 35.6%), and dementia (*n* = 185, 26.9%). Although the median number of years of education was identical across the groups (12 years), the distribution differed significantly (*p* < 0.001). No significant differences were observed in the prevalence of a family history of dementia. Of the 688 participants, 186 (26.6%) underwent GA after the age of 50 years. Between-group comparisons showed that age, education level, diabetes mellitus, alcohol consumption, and APOE ε4 carrier status differed significantly across normal cognition, MCI, and dementia groups (all *p* < 0.05). Overall, 258 participants (37.5%) were APOE ε4 carriers and 324 (47.1%) were amyloid PET-positive. The proportions of APOE ε4 carriers and amyloid PET-positive participants were highest in the dementia group (both *p* < 0.001).

### 3.2. Variables Associated with CDR-SB and GDS Scores

Table 3 and Table 4 present the results of the logistic regression analyses for CDR-SB and GDS, respectively. In the cognitively normal group, GA exposure remained significantly associated with both higher CDR-SB (OR = 2.33 [95% CI: 1.06–5.08, *p* = 0.032]) and GDS scores (OR = 2.29 [95% CI: 1.16–4.55, *p* = 0.017]) after multivariable adjustment. The comorbidity count and amyloid PET positivity were also significantly associated with both outcomes. In contrast, GA exposure was significantly associated with CDR-SB but not GDS in the MCI group, whereas no significant association was observed in the dementia group. Sensitivity analyses using the three-category classification yielded similar overall patterns, although the association between GA exposure and CDR-SB in the MCI group was not reproduced, and some subgroup estimates were less stable because of the small number of participants in each category (Tables S2 and S3). Furthermore, exploratory interaction analyses were performed according to APOE ε4 carrier status and amyloid PET positivity (Table S4). No consistent interaction patterns were observed across diagnostic groups.

### 3.3. Variables Associated with Amyloid PET SUVR

Table 5 summarizes the linear regression analyses of the variables associated with amyloid PET SUVR. GA exposure was significantly associated with higher SUVRs in both the univariate (β = 0.13, 95% CI: 0.04–0.22, *p* = 0.007) and multivariable analyses (β = 0.09, 95% CI: 0.004–0.18, *p* = 0.040). Age, years of education, and APOE ε4 carrier status were also independently associated with SUVR.

### 3.4. Comparison of CDR-SB and GDS Scores According to GA Exposure

**Figure 2:**
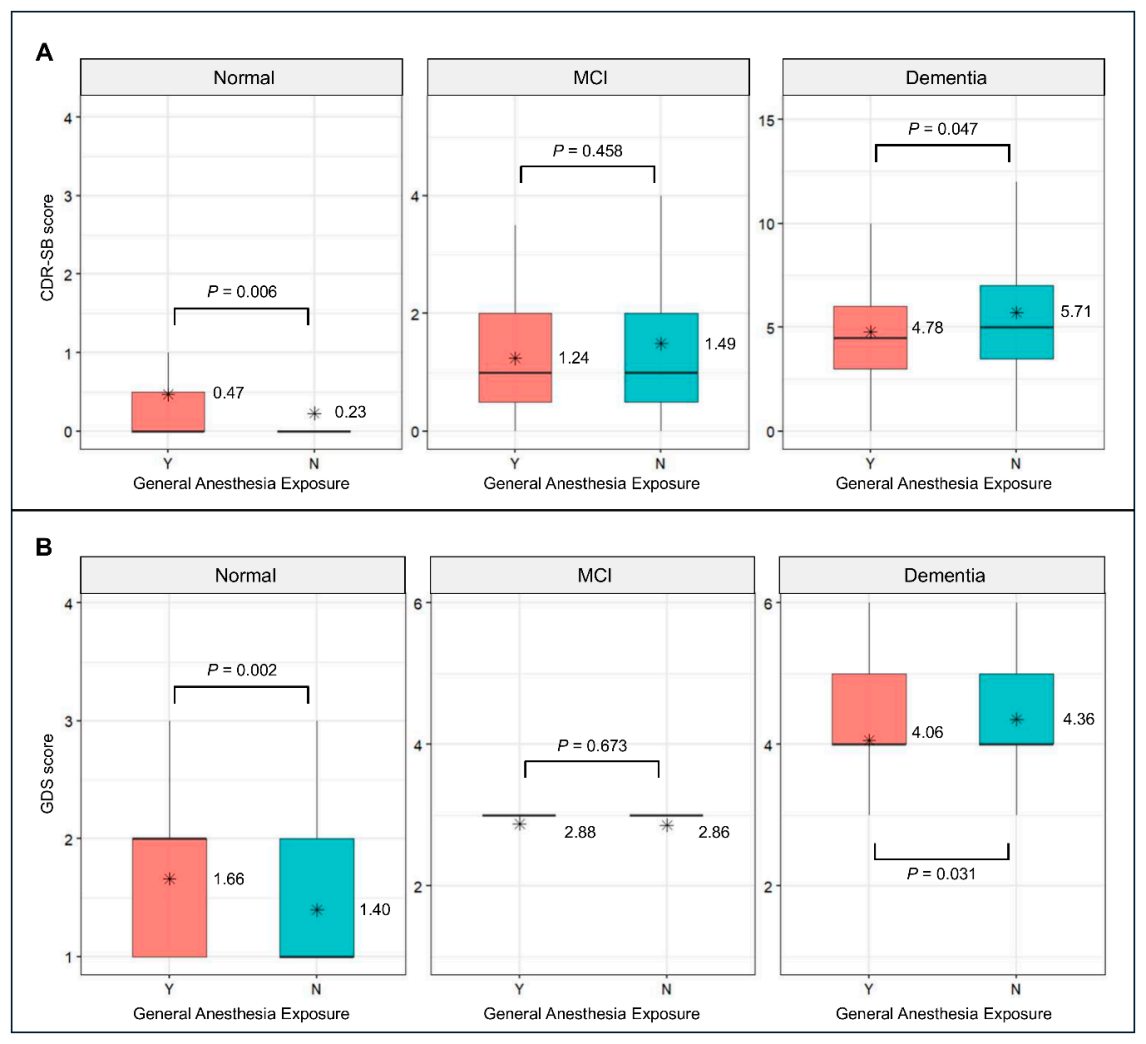
Comparison of CDR-SB (**A**) and GDS (**B**) scores between participants with (Y) and without (N) general anesthesia exposure, stratified by diagnostic group. Box plots display the median (horizontal line), interquartile range (box), and mean (asterisk) values. Abbreviations: MCI, mild cognitive impairment; CDR-SB, Clinical Dementia Rating Sum of Boxes; GDS, Global Deterioration Scale.

Figure 2 shows unadjusted comparisons of the CDR-SB and GDS scores according to GA exposure within each diagnostic group. In the normal group, participants with GA exposure had significantly higher CDR-SB and GDS scores than those without GA exposure (0.47 vs. 0.23 for CDR-SB, 1.66 vs. 1.40 for GDS; all, *p* < 0.05). No significant differences were observed in the MCI group. In contrast, in the dementia group, participants with GA exposure had lower unadjusted CDR-SB and GDS scores than those without GA exposure; however, these differences were not significant in the multivariable analyses (Table 3 and Table 4).

## 4. Discussion

In this cross-sectional study, we examined whether GA exposure after the age of 50 years was associated with cognitive severity, as measured by CDR-SB and GDS scores, across different diagnostic groups. In the cognitively normal group, GA exposure was independently associated with higher CDR-SB and GDS scores after multivariable adjustment. GA exposure was also independently associated with a higher amyloid PET SUVR. In contrast, we did not observe a consistent association in participants with MCI. Although GA exposure was associated with CDR-SB in the primary analysis, it was not replicated in the sensitivity analysis using the three-category classification (low, intermediate, and high), and no significant association was observed for GDS or amyloid PET SUVR. No significant association was observed in participants with dementia.

While CDR-SB and GDS differed significantly according to GA exposure, the absolute difference was small (mean CDR-SB, 0.47 vs. 0.23; mean GDS, 1.66 vs. 1.40). However, the overall pattern of findings was consistent across the two cognitive severity measures in the multivariable analyses, sensitivity analyses using the three severity categories, and amyloid PET SUVR. Moreover, both the CDR-SB and GDS are subject to marked floor effects in cognitively normal individuals. Therefore, even small differences may reflect subtle but meaningful differences in cognitive function.

Several mechanisms may explain this observed association, although causality cannot be established given the cross-sectional nature of this study. Previous studies have suggested that GA may be associated with cognitive impairment via neuroinflammation, disruption of the blood–brain barrier, structural changes, or exacerbation of neurodegenerative processes [8,13]. These effects may vary according to the anesthetic agent, depth of anesthesia, and perioperative course [1]. Conversely, reverse causation cannot be excluded. Individuals with subtle preclinical cognitive decline may be more likely to undergo surgery requiring GA because of early gait impairment, frailty, or other functional declines associated with future cognitive decline [23,24,25]. Alternatively, underlying diseases requiring surgery, the surgical process itself, or postoperative factors such as reduced physical activity, may be associated with cognitive function.

The inconsistent findings in the MCI group may reflect the heterogeneous and transitional nature of this stage in which individuals differ widely in the degree of underlying neuropathology and cognitive vulnerability. Similarly, no significant associations were observed in the dementia group. Advanced neurodegeneration and the varied clinical characteristics of established dementia may obscure any modest associations with GA exposure. Patients with dementia who undergo surgery represent a clinically heterogeneous population. Some patients undergo surgery because of falls, fractures, or complications related to functional decline, whereas others are less likely to undergo surgery because of advanced disease or frailty. This heterogeneity may have diluted the underlying associations.

This study has several limitations. First, detailed information on GA exposure was unavailable. GA exposure was defined as a self-reported binary variable (yes/no) after the age of 50 years, without information on anesthetic agents, the duration and number of GA exposures, surgical indications and types, perioperative management, or complications. All of these factors can independently influence cognitive outcomes. This simplified definition may have reduced the recall burden for detailed perioperative information, but limited our ability to examine this association in detail. Second, because of the cross-sectional design, we could not determine whether the observed associations reflected the effects of GA itself, the surgical procedure, the underlying condition requiring surgery, or perioperative factors. Third, a temporal relationship between GA exposure and cognitive decline could not be determined. Although baseline cognitive status is less likely to influence eligibility for surgery among cognitively normal individuals than among patients with MCI or dementia, selection bias cannot be completely excluded. Fourth, residual confounding factors cannot be excluded despite adjustment for multiple covariates. Factors such as frailty, socioeconomic status, and lifestyle may have influenced the observed associations. In addition, survivor bias is possible because individuals with poorer postoperative outcomes may have been less likely to survive until enrollment in the registry. Finally, although the overall sample size was relatively large, the analyses within each diagnostic group resulted in smaller subgroup sample sizes. Some estimates were imprecise, as reflected by the relatively wide confidence intervals, and modest associations may have been missed because of limited statistical power. Therefore, our findings should be interpreted with caution and validated in larger prospective studies.

Despite these limitations, this study has several strengths. Cognitive diagnoses were established by specialists based on comprehensive neurocognitive assessments and neuroimaging findings. APOE ε4 carrier status and amyloid PET biomarkers were also available for analyses. In addition, stratification by diagnostic group allowed us to evaluate the association between GA exposure and cognitive severity among participants with similar cognitive status. These consistent findings across different cognitive measures and amyloid PET SUVRs further support the robustness of our results.

## 5. Conclusions

Our findings suggest that GA exposure may be associated with greater cognitive burden in cognitively normal individuals. Future prospective longitudinal studies incorporating detailed anesthetic information, surgical characteristics, perioperative complications, and neurodegenerative biomarkers are required to clarify this relationship.

## Figures and Tables

**Table 1:** Severity thresholds and distribution of CDR-SB and GDS categories by GA exposure across diagnostic groups.

Group	Category	Threshold	GA− (*n*)	GA+ (*n*)	Total (*n*)
	CDR-SB				
Normal	Low	0	159	37	196
	High	≥0.5	40	22	62
MCI	Low	≤2.0	131	64	195
	High	≥2.5	43	7	50
Dementia	Low	≤9.0	114	47	161
	High	≥9.5	18	6	24
	GDS				
Normal	Low	1	139	28	167
	High	≥2	58	31	89
MCI	Low	≤3	156	65	221
	High	≥4	17	3	20
Dementia	Low	≤4	76	36	112
	High	≥5	55	15	70

Abbreviations: MCI, mild cognitive impairment; CDR-SB, Clinical Dementia Rating Sum of Boxes; GDS, Global Deterioration Scale; GA, general anesthesia.

**Table 2:** Demographic, clinical, and neuroimaging characteristics of study participants among the different diagnostic groups.

	Total Participants (*N* = 688)			*p*-Value
	Normal (*n* = 258, 37.5%)	MCI (*n* = 245, 35.6%)	Dementia (*n* = 185, 26.9%)	
Demographics				
Age, years (Median (IQR])	73 (66–77)	74 (67–79)	74 (67–80)	0.013
Female, *n* (%)	156 (60.5%)	156 (63.7%)	121 (65.4%)	0.544
Education, years (Median (IQR))	12 (9.0–16.0)	12 (6.0–16.0)	12 (6.0–14.0)	<0.001
Family history of dementia, *n* (%)	77 (29.8%)	88 (35.9%)	61 (33.0%)	0.349
Medical history				
GA exposure, *n* (%)	59 (22.9%)	71 (29.0%)	53 (28.6%)	0.229
Ever smoking, *n* (%)	75 (29.1)	55 (22.5)	47 (25.4)	0.245
History of alcohol consumption, *n* (%)	116 (45.0)	100 (41.0)	59 (31.9)	0.020
Diabetes mellitus, *n* (%)	37 (14.3%)	59 (24.1%)	39 (21.1%)	0.019
Hypertension, *n* (%)	132 (51.2%)	100 (40.8%)	81 (43.8%)	0.057
Dyslipidemia, *n* (%)	115 (44.6%)	113 (46.1%)	66 (35.7%)	0.072
Comorbidity count, mean (SD)	0.32 (0.54)	0.42 (0.66)	0.37 (0.59)	0.127
Clinical assessment measures (Median [IQR])				
MMSE, score	28 (27.0–29.0)	26 (23.0–28.0)	18 (15.0–22.0)	<0.001
MMSE, Z-score	0.46 (−0.14–1.02)	−0.75 (−1.90–0.08)	−4.26 (−6.63–−2.21)	<0.001
CDR	0.0 (0.0–0.0)	0.5 (0.5–0.5)	1.0 (0.5–1.0)	<0.001
CDR-SB	0.0 (0.0–0.0)	1.0 (0.5–2.0)	5.0 (3.5–7.0)	<0.001
GDS ^a^	1.0 (1.0–2.0)	3.0 (3.0–3.0)	4.0 (4.0–5.0)	<0.001
Biomarkers				
APOE ε4 carrier	48 (18.6%)	113 (46.1%)	97 (52.4%)	<0.001
Positive for amyloid PET	45 (17.4%)	129 (52.7%)	150 (81.1%)	<0.001
SUVR (Median (IQR))	0.64 (0.56–0.92)	1.06 (0.78–1.37)	1.37 (1.13–1.56)	<0.001

^a^ GDS data were unavailable for nine participants who were excluded from the GDS-related analysis. Abbreviations: MCI, mild cognitive impairment; IQR, interquartile range; GA, general anesthesia; APOE, apolipoprotein E; MMSE, Mini-Mental State Examination; CDR-SB, Clinical Dementia Rating Sum of Boxes; GDS, Global Deterioration Scale; PET, positron emission tomography; SUVR, standardized uptake value ratio.

**Table 3:** Logistic regression analysis of variables associated with CDR-SB scores across diagnostic groups.

Variables	Univariate Analysis		Multivariable Analysis	
	OR (95% CI)	*p*-Value	OR (95% CI)	*p*-Value
**Normal**				
Age	1.03 (0.99–1.07)	0.135	1.01 (0.96–1.06)	0.798
Sex (female)	1.66 (0.91–3.11)	0.102	7.15 (1.59–43.59)	0.018
Education, year	0.91 (0.86–0.97)	0.004	0.96 (0.89–1.04)	0.338
Family history of dementia	1.41 (0.76–2.57)	0.267	1.42 (0.67–3.00)	0.353
GA exposure	2.36 (1.25–4.43)	0.008	2.33 (1.07–5.09)	0.032
Ever smoking	1.10 (0.58–2.04)	0.754	8.15 (1.86–47.04)	0.010
History of alcohol consumption	0.50 (0.27–0.90)	0.022	0.51 (0.19–1.31)	0.171
Diabetes mellitus	1.02 (0.43–2.22)	0.964	0.86 (0.31–2.23)	0.767
Hypertension	1.11 (0.63–1.98)	0.709	0.55 (0.25–1.17)	0.127
Dyslipidemia	1.72 (0.97–3.08)	0.064	1.82 (0.87–3.88)	0.114
Comorbidity count	2.27 (1.37–3.81)	0.002	2.67 (1.46–5.06)	0.002
APOE ε4 carrier	1.78 (0.88–3.50)	0.097	0.93 (0.35–2.33)	0.881
PET-amyloid+	8.67 (4.33–17.88)	<0.001	9.34 (4.02–22.93)	<0.001
**MCI**				
Age	1.04 (1.00–1.09)	0.074	1.04 (0.98–1.10)	0.178
Sex (female)	1.82 (0.93–3.76)	0.092	1.20 (0.37–4.22)	0.763
Education, year	0.92 (0.86–0.98)	0.011	0.92 (0.85–0.99)	0.028
Family history of dementia	0.80 (0.41–1.54)	0.518	0.77 (0.35–1.64)	0.499
GA exposure	0.33 (0.13–0.74)	0.012	0.23 (0.08–0.56)	0.002
Ever smoking	0.61 (0.25–1.34)	0.248	1.01 (0.28–3.69)	0.994
History of alcohol consumption	0.64 (0.32–1.22)	0.187	0.62 (0.24–1.53)	0.304
Diabetes mellitus	1.30 (0.63–2.57)	0.468	1.17 (0.48–2.77)	0.730
Hypertension	1.60 (0.85–3.00)	0.141	0.99 (0.44–2.22)	0.986
Dyslipidemia	1.49 (0.80–2.79)	0.212	1.92 (0.88–4.28)	0.102
Comorbidity count	0.95 (0.57–1.52)	0.840	1.11 (0.58–2.02)	0.739
APOE ε4 carrier	1.82 (0.98–3.46)	0.061	1.41 (0.61–3.28)	0.420
PET-amyloid+	4.14 (2.06–8.92)	<0.001	5.02 (2.11–12.93)	<0.001
**Dementia**				
Age	1.00 (0.95–1.05)	0.887	1.00 (0.95–1.06)	0.865
Sex (female)	1.69 (0.67–4.87)	0.294	1.62 (0.41–7.32)	0.506
Education, year	1.00 (0.92–1.08)	0.915	0.99 (0.89–1.11)	0.906
Family history of dementia	0.64 (0.22–1.63)	0.376	0.82 (0.26–2.28)	0.709
GA exposure	0.81 (0.28–2.06)	0.672	0.86 (0.27–2.40)	0.776
Ever smoking	0.55 (0.15–1.55)	0.298	0.90 (0.17–4.40)	0.903
History of alcohol consumption	0.68 (0.24–1.73)	0.440	0.95 (0.28–2.91)	0.932
Diabetes mellitus	0.98 (0.31–2.65)	0.975	1.40 (0.40–4.31)	0.575
Hypertension	0.60 (0.23–1.45)	0.272	0.62 (0.21–1.67)	0.355
Dyslipidemia	0.56 (0.19–1.42)	0.247	0.67 (0.22–1.84)	0.453
Comorbidity count	0.74 (0.30–1.56)	0.467	1.02 (0.38–2.44)	0.971
APOE ε4 carrier	1.08 (0.46–2.60)	0.855	0.85 (0.34–2.16)	0.732
PET-amyloid+	6.16 (1.23–112.12)	0.080	6.44 (1.15–122.22)	0.085

Abbreviations: CDR-SB, Clinical Dementia Rating Sum of Boxes; OR, odds ratio; CI, confidence interval; MCI, mild cognitive impairment; GA, general anesthesia; APOE ε4, E4 variant of apolipoprotein E; PET, positron emission tomography.

**Table 4:** Logistic regression analysis of variables associated with GDS scores across diagnostic groups.

Variables	Univariate Analysis		Multivariable Analysis	
	OR (95% CI)	*p*-Value	OR (95% CI)	*p*-Value
**Normal**				
Age	1.04 (1.01–1.08)	0.024	1.02 (0.97–1.06)	0.442
Sex (female)	1.69 (0.99–2.94)	0.057	3.01 (0.99–10.39)	0.064
Education, year	0.91 (0.86–0.97)	0.002	0.95 (0.89–1.02)	0.171
Family history of dementia	1.30 (0.74–2.25)	0.356	1.39 (0.73–2.63)	0.310
GA exposure	2.65 (1.46–4.84)	0.001	2.29 (1.16–4.55)	0.017
Ever smoking	0.79 (0.44–1.40)	0.430	1.73 (0.57–5.70)	0.347
History of alcohol consumption	0.61 (0.36–1.03)	0.066	0.99 (0.46–2.13)	0.976
Diabetes mellitus	1.52 (0.74–3.09)	0.244	1.44 (0.63–3.24)	0.386
Hypertension	1.42 (0.85–2.40)	0.180	0.85 (0.45–1.61)	0.623
Dyslipidemia	1.52 (0.91–2.56)	0.113	1.12 (0.60–2.10)	0.718
Comorbidity count	2.48 (1.53–4.12)	<0.001	2.45 (1.42–4.36)	0.002
APOE ε4 carrier	1.29 (0.67–2.45)	0.438	0.75 (0.32–1.68)	0.498
PET-amyloid+	5.28 (2.66–10.96)	<0.001	4.72 (2.13–10.93)	<0.001
**MCI**				
Age	1.02 (0.96–1.09)	0.549	1.00 (0.92–1.08)	0.988
Sex (female)	1.10 (0.43–3.02)	0.852	3.82 (0.53–39.62)	0.216
Education, year	1.01 (0.93–1.12)	0.763	1.03 (0.92–1.16)	0.619
Family history of dementia	1.53 (0.59–3.86)	0.367	1.25 (0.41–3.78)	0.694
GA exposure	0.42 (0.10–1.31)	0.182	0.36 (0.07–1.30)	0.152
Ever smoking	1.62 (0.55–4.33)	0.353	6.71 (0.93–75.28)	0.084
History of alcohol consumption	0.62 (0.21–1.64)	0.356	0.37 (0.08–1.46)	0.175
Diabetes mellitus	0.53 (0.12–1.66)	0.329	0.36 (0.05–1.60)	0.224
Hypertension	1.85 (0.74–4.76)	0.192	2.65 (0.78–9.46)	0.121
Dyslipidemia	0.47 (0.16–1.23)	0.140	0.38 (0.11–1.21)	0.115
Comorbidity count	1.19 (0.58–2.19)	0.609	1.57 (0.63–3.61)	0.305
APOE ε4 carrier	3.84 (1.43–12.13)	0.012	2.76 (0.78–11.74)	0.135
PET-amyloid+	9.42 (2.63–60.14)	0.003	6.29 (1.43–45.35)	0.029
**Dementia**				
Age	0.98 (0.95–1.01)	0.230	0.99 (0.95–1.03)	0.598
Sex (female)	1.94 (1.02–3.80)	0.048	4.03 (1.39–12.87)	0.013
Education, year	1.02 (0.96–1.08)	0.594	1.04 (0.97–1.13)	0.282
Family history of dementia	0.72 (0.37–1.36)	0.320	0.98 (0.47–2.06)	0.956
GA exposure	0.58 (0.28–1.14)	0.120	0.56 (0.25–1.21)	0.147
Ever smoking	0.71 (0.35–1.42)	0.346	1.83 (0.59–6.11)	0.307
History of alcohol consumption	0.70 (0.36–1.33)	0.281	0.93 (0.40–2.12)	0.854
Diabetes mellitus	1.48 (0.71–3.06)	0.296	2.53 (1.09–6.07)	0.033
Hypertension	0.54 (0.29–1.00)	0.051	0.56 (0.27–1.14)	0.114
Dyslipidemia	0.82 (0.43–1.52)	0.525	0.71 (0.34–1.45)	0.347
Comorbidity count	0.80 (0.47–1.34)	0.414	1.13 (0.61–2.07)	0.700
APOE ε4 carrier	0.54 (0.29–0.99)	0.047	0.40 (0.20–0.79)	0.010
PET-amyloid+	1.85 (0.83–4.45)	0.148	2.58 (1.01–7.15)	0.056

GDS, Global Deterioration Scale; OR, odds ratio; CI, confidence interval; MCI, mild cognitive impairment; GA, general anesthesia; APOE, apolipoprotein E; PET, positron emission tomography.

**Table 5:** Linear regression analysis of variables associated with SUVRs among the different diagnostic groups.

Variables	Univariate Analysis		Multivariable Analysis	
	β (95% CI)	*p*-Value	β (95% CI)	*p*-Value
**Normal**				
Age	0.01 (0.00–0.01)	0.001	0.01 (0.00–0.01)	0.008
Sex (female)	−0.02 (−0.10–0.06)	0.702	0.06 (−0.06–0.18)	0.315
Education, year	−0.01 (−0.02–−0.01)	0.001	−0.01 (−0.02–0.00)	0.018
Family history of dementia	0.03 (−0.06–0.11)	0.554	0.04 (−0.04–0.12)	0.347
GA exposure	0.13 (0.04–0.22)	0.007	0.09 (0.00–0.18)	0.040
Ever smoking	0.08 (−0.01–0.17)	0.069	0.17 (0.05–0.29)	0.005
History of alcohol consumption	−0.02 (−0.10–0.05)	0.535	−0.05 (−0.14–0.04)	0.258
Diabetes mellitus	0.01 (−0.10–0.12)	0.855	−0.04 (−0.14–0.07)	0.509
Hypertension	0.04 (−0.04–0.11)	0.372	−0.03 (−0.11–0.04)	0.389
Dyslipidemia	0.03 (−0.04–0.11)	0.391	−0.02 (−0.10–0.06)	0.610
Comorbidity count	0.09 (0.02–0.16)	0.016	0.07 (0.00–0.14)	0.065
APOE ε4 carrier	0.12 (0.02–0.22)	0.017	0.13 (0.04–0.22)	0.007
**MCI**				
Age	0.00 (0.00–0.01)	0.691	0.00 (0.00–0.01)	0.153
Sex (female)	−0.02 (−0.12–0.08)	0.640	−0.01 (−0.15–0.13)	0.877
Education, year	0.00 (−0.01–0.01)	0.750	0.00 (−0.01–0.01)	0.801
Family history of dementia	0.11 (0.01–0.21)	0.030	0.07 (−0.03–0.17)	0.152
GA exposure	0.04 (−0.07–0.14)	0.487	0.02 (−0.09–0.12)	0.747
Ever smoking	−0.01 (−0.12–0.11)	0.924	−0.06 (−0.21–0.09)	0.443
History of alcohol consumption	0.02 (−0.08–0.12)	0.659	0.04 (−0.07–0.15)	0.513
Diabetes mellitus	−0.02 (−0.13–0.09)	0.694	0.01 (−0.10–0.12)	0.880
Hypertension	0.03 (−0.07–0.12)	0.594	0.05 (−0.06–0.15)	0.368
Dyslipidemia	−0.01 (−0.11–0.08)	0.767	−0.05 (−0.15–0.05)	0.331
Comorbidity count	0.03 (−0.04–0.10)	0.420	0.00 (−0.07–0.08)	0.974
APOE ε4 carrier	0.32 (0.23–0.41)	<0.001	0.33 (0.24–0.42)	<0.001
**Dementia**				
Age	0.00 (−0.01–0.00)	0.146	0.00 (−0.01–0.00)	0.669
Sex (female)	−0.02 (−0.12–0.08)	0.668	0.02 (−0.13–0.17)	0.774
Education, year	0.01 (0.00–0.02)	0.003	0.01 (0.00–0.03)	0.009
Family history of dementia	−0.10 (−0.20–0.00)	0.058	−0.15 (−0.25–−0.04)	0.006
GA exposure	−0.01 (−0.12–0.09)	0.829	0.06 (−0.05–0.17)	0.274
Ever smoking	0.01 (−0.10–0.12)	0.907	0.00 (−0.15–0.16)	0.968
History of alcohol consumption	−0.01 (−0.11–0.09)	0.806	0.00 (−0.11–0.12)	0.980
Diabetes mellitus	−0.14 (−0.25–−0.03)	0.017	−0.13 (−0.25–−0.02)	0.026
Hypertension	−0.07 (−0.17–0.02)	0.141	−0.01 (−0.11–0.09)	0.842
Dyslipidemia	−0.03 (−0.13–0.07)	0.504	0.01 (−0.09–0.11)	0.786
Comorbidity count	−0.08 (−0.16–0.00)	0.050	−0.07 (−0.15–0.01)	0.095
APOE ε4 carrier	0.08 (−0.01–0.18)	0.080	0.09 (0.00–0.18)	0.063

Abbreviations: β, unstandardized regression coefficient; CI, confidence interval; MCI, mild cognitive impairment; GA, general anesthesia; APOE, apolipoprotein E; PET, positron emission tomography.

## Data Availability

The data underlying this article are available from the corresponding author upon reasonable request.

## Supplementary Materials

The following supporting information can be downloaded at: https://www.mdpi.com/article/10.3390/jcm15155864/s1, Figure S1. Raw distribution of CDR-SB by diagnostic group (Normal, MCI, Dementia). Histogram plots display the empirical density of CDR-SB values separately for each diagnostic category.; Figure S2: Raw distribution of GDS scores within each diagnostic group (Normal, MCI, Dementia); Table S1. CDR-SB and GDS severity thresholds and distribution by GA exposure (3-category); Table S2. Logistic regression results using three-category CDR-SB classifications by diagnostic groups; Table S3. Logistic regression results using three-category GDS classifications by diagnostic groups; Table S4. Effect modification of the association between GA exposure and cognitive severity by APOE ε4 carrier status and amyloid positivity.

## Abbreviations

The following abbreviations are used in this manuscript:

APOE ε4	Apolipoprotein E ε4 allele
CDR	Clinical Dementia Rating
CDR-SB	Clinical Dementia Rating Sum of Boxes
GA	General anesthesia
GDS	Global Deterioration Scale
KDRC TRR	Korea Dementia Research Center’s Trial Ready Registry
MCI	Mild cognitive impairment
MRI	Magnetic Resonance Imaging
NIA-AA	National Institute on Aging–Alzheimer’s Association
PET	Positron Emission Tomography
SUVRs	Standardized uptake value ratios
