# Supplementary material for: Association of General Anesthesia Exposure with Cognitive Outcomes in Older Adults: A Cross-Sectional Study in a Korean Cohort
Source: J Clin Med. 2026 Jul 27;15(15):5864. doi: 10.3390/jcm15155864 (PMC13467552; doi:10.3390/jcm15155864)
Supplement: Supplementary file 1 [file jcm-15-05864-s001.zip › jcm-4418381-supplementary.pdf]

Supplementary Materials

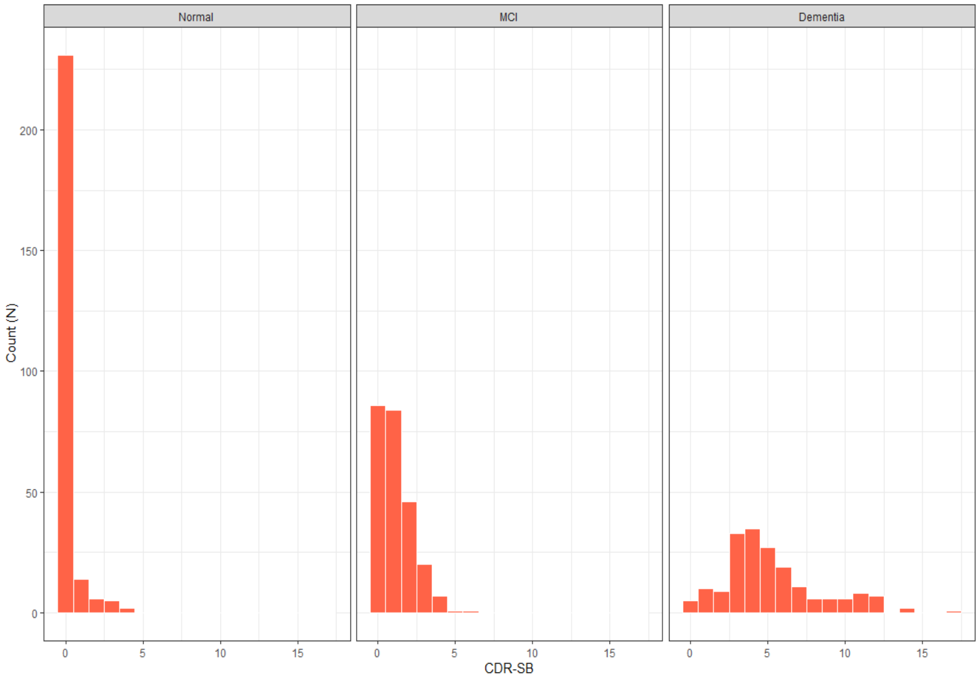

Figure S1. Raw distribution of CDR-SB by diagnostic group (Normal, MCI, Dementia). Histogram plots display the empirical density of CDR-SB values separately for each diagnostic category. Abbreviations: CDR-SB, Clinical Dementia Rating Sum of Boxes; MCI, mild cognitive impairment.

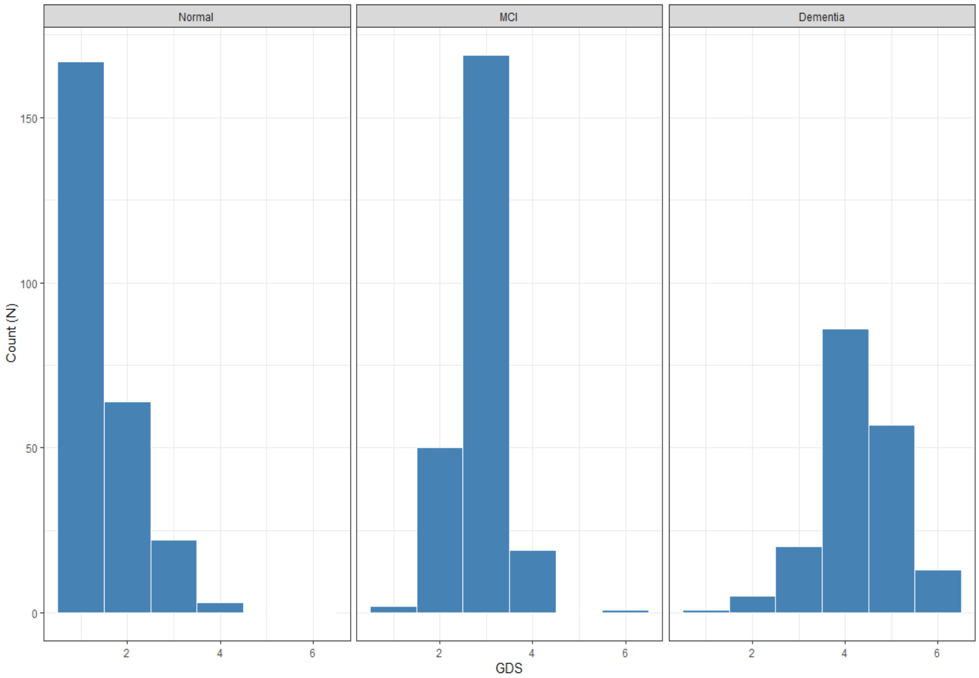

Figure S2. Raw distribution of GDS scores within each diagnostic group (Normal, MCI, Dementia). Abbreviations: GDS, Global Deterioration Scale; MCI, mild cognitive impairment.

Table S1. CDR-SB and GDS severity thresholds and distribution by GA exposure (3-category).

Group	Category	Threshold	GA− (*n*)	GA+ (*n*)	Total (*n*)
	CDR-SB				
Normal	Low	0	159	37	196
	Intermediate	0.5	23	12	35
	High	>0.5	17	10	27
MCI	Low	≤0.5	64	22	86
	Intermediate	>0.5–2.0	67	42	109
	High	≥2.5	43	7	50
Dementia	Low	≤4.0	49	23	72
	Intermediate	4.5–9.0	65	24	89
	High	≥9.5	18	6	24
	GDS				
Normal	Low	1	139	28	167
	Intermediate	2	40	24	64
	High	>2	18	7	25
MCI	Low	≤2	41	11	52
	Intermediate	3	115	54	169
	High	>3	17	3	20
Dementia	Low	≤3	14	12	26
	Intermediate	4–5	106	37	143
	High	>5	11	2	13

GA, general anesthesia; CDR-SB, Clinical Dementia Rating Sum of Boxes; GDS, Global Deterioration Scale. Thresholds were defined based on prior literature. GA−, no GA exposure; GA+, GA exposure. GDS was missing in 9 participants.

Table S2. Logistic regression results using three-category CDR-SB classifications by diagnostic groups.

Variables	Univariate Analysis		Multivariate Analysis	
	OR (95% CI)	*p*-Value	OR (95% CI)	*p*-Value
**Normal**				
Age	1.03 (1.00–1.08)	0.084	1.02 (0.97–1.07)	0.551
Sex (female)	1.69 (0.94–3.16)	0.088	7.44 (1.77–36.72)	0.009
Education, year	0.91 (0.85–0.96)	0.002	0.96 (0.89–1.03)	0.286
Family history of dementia	1.52 (0.83–2.77)	0.170	1.61 (0.79–3.21)	0.182
GA exposure	2.33 (1.24–4.31)	0.007	2.07 (1.00–4.26)	0.048
Ever smoking, *n* (%)	1.04 (0.56–1.91)	0.892	6.90 (1.75–31.57)	0.008
History of alcohol consumption, *n* (%)	0.48 (0.26–0.87)	0.017	0.62 (0.25–1.49)	0.299
Diabetes mellitus	1.16 (0.49–2.54)	0.717	1.09 (0.41–2.69)	0.850
Hypertension	1.15 (0.65–2.03)	0.630	0.60 (0.29–1.20)	0.148
Dyslipidemia	1.82 (1.03–3.24)	0.039	2.02 (1.01–4.13)	0.049
Comorbidity counts	2.03 (1.27–3.25)	0.003	2.17 (1.25–3.78)	0.006
APOE ε4 carrier	1.89 (0.94–3.67)	0.065	0.98 (0.40–2.29)	0.970
PET-amyloid+	8.58 (4.43–16.88)	<0.001	8.34 (3.80–18.87)	<0.001
**MCI**				
Age	1.04 (1.01–1.07)	0.017	1.03 (1.00–1.07)	0.077
Sex (female)	1.34 (0.82–2.17)	0.242	1.11 (0.49–2.50)	0.807
Education, year	0.93 (0.89–0.98)	0.004	0.91 (0.86–0.97)	0.002
Family history of dementia	1.29 (0.80–2.09)	0.301	1.21 (0.71–2.07)	0.482
GA exposure	0.85 (0.51–1.40)	0.517	0.59 (0.34–1.04)	0.072
Ever smoking, *n* (%)	0.77 (0.44–1.34)	0.355	0.83 (0.36–1.89)	0.648
History of alcohol consumption, *n* (%)	0.75 (0.47–1.21)	0.240	0.89 (0.47–1.69)	0.714
Diabetes mellitus	1.02 (0.58–1.78)	0.945	1.14 (0.61–2.15)	0.675
Hypertension	1.20 (0.74–1.94)	0.455	0.88 (0.49–1.55)	0.653
Dyslipidemia	0.99 (0.62–1.59)	0.981	1.03 (0.59–1.79)	0.920
Comorbidity counts	1.11 (0.78–1.58)	0.568	1.31 (0.87–1.97)	0.195
APOE ε4 carrier	1.95 (1.22–3.16)	0.006	1.37 (0.76–2.46)	0.294
PET-amyloid+	3.98 (2.42–6.65)	<0.001	4.48 (2.49–8.22)	<0.001
**Dementia**				
Age	1.01 (0.98–1.04)	0.636	1.01 (0.98–1.05)	0.487
Sex (female)	1.95 (1.09–3.54)	0.026	1.94 (0.73–5.20)	0.184
Education, year	0.95 (0.90–1.01)	0.078	0.96 (0.89–1.03)	0.246
Family history of dementia	0.65 (0.36–1.16)	0.147	0.88 (0.46–1.68)	0.704
GA exposure	0.78 (0.42–1.43)	0.421	0.82 (0.42–1.58)	0.555
Ever smoking, *n* (%)	0.63 (0.33–1.18)	0.149	1.32 (0.47–3.73)	0.598
History of alcohol consumption, *n* (%)	0.66 (0.36–1.20)	0.174	0.80 (0.38–1.68)	0.559
Diabetes mellitus	1.12 (0.57–2.20)	0.736	1.48 (0.71–3.11)	0.295
Hypertension	0.52 (0.30–0.91)	0.024	0.45 (0.24–0.84)	0.014
Dyslipidemia	0.74 (0.41–1.31)	0.298	0.74 (0.39–1.38)	0.342
Comorbidity counts	1.07 (0.68–1.70)	0.762	1.44 (0.87–2.42)	0.159
APOE ε4 carrier	0.58 (0.33–1.00)	0.053	0.49 (0.27–0.90)	0.022
PET-amyloid+	1.35 (0.68–2.69)	0.388	2.02 (0.92–4.51)	0.082

Abbreviations: CDR-SB, clinical dementia rating sum of boxes; OR, odds ratio; CI, confidential interval; MCI, mild cognitive impairment; GA, general anesthesia; PET, positron emission tomography.

Table S3. Logistic regression results using three-category GDS classifications by diagnostic groups.

Variables	Univariate Analysis		Multivariate Analysis	
	OR (95% CI)	*p*-Value	OR (95% CI)	*p*-Value
**Normal**				
Age	1.04 (1.01–1.08)	0.016	1.02 (0.98–1.07)	0.272
Sex (female)	1.66 (0.98–2.86)	0.063	2.71 (0.94–8.69)	0.077
Education, year	0.91 (0.86–0.96)	0.001	0.96 (0.90–1.02)	0.174
Family history of dementia	1.34 (0.78–2.30)	0.287	1.45 (0.79–2.62)	0.222
GA exposure	2.34 (1.32–4.11)	0.003	1.97 (1.05–3.70)	0.034
Ever smoking, *n* (%)	0.79 (0.44–1.38)	0.413	1.49 (0.53–4.50)	0.461
History of alcohol consumption, *n* (%)	0.62 (0.37–1.03)	0.068	1.09 (0.52–2.27)	0.823
Diabetes mellitus	1.70 (0.83–3.39)	0.135	1.61 (0.74–3.43)	0.220
Hypertension	1.43 (0.86–2.39)	0.169	0.81 (0.44–1.48)	0.496
Dyslipidemia	1.69 (1.02–2.82)	0.044	1.35 (0.75–2.44)	0.314
Comorbidity counts	2.01 (1.30–3.13)	0.002	1.78 (1.09–2.91)	0.022
APOE ε4 carrier	1.55 (0.81–2.93)	0.180	0.88 (0.40–1.88)	0.750
PET-amyloid+	6.79 (3.50–13.46)	<0.001	6.01 (2.84–13.01)	<0.001
**MCI**				
Age	1.02 (0.98–1.05)	0.385	1.00 (0.96–1.04)	0.829
Sex (female)	1.01 (0.57–1.77)	0.973	1.20 (0.48–3.01)	0.702
Education, year	1.00 (0.94–1.05)	0.882	0.98 (0.92–1.05)	0.640
Family history of dementia	1.39 (0.78–2.49)	0.269	1.07 (0.57–2.04)	0.825
GA exposure	1.13 (0.62–2.07)	0.693	0.95 (0.49–1.86)	0.882
Ever smoking, *n* (%)	1.03 (0.54–2.02)	0.922	1.52 (0.58–3.99)	0.391
History of alcohol consumption, *n* (%)	0.75 (0.43–1.30)	0.302	0.60 (0.29–1.25)	0.173
Diabetes mellitus	1.10 (0.59–2.08)	0.776	1.09 (0.54–2.22)	0.809
Hypertension	1.79 (1.02–3.20)	0.046	1.88 (0.97–3.69)	0.065
Dyslipidemia	1.09 (0.63–1.89)	0.759	0.80 (0.42–1.52)	0.491
Comorbidity counts	1.42 (0.93–2.17)	0.105	1.42 (0.88–2.33)	0.158
APOE ε4 carrier	3.77 (2.07–7.15)	<0.001	2.21 (1.09–4.63)	0.031
PET-amyloid+	7.16 (3.71–14.83)	<0.001	5.64 (2.70–12.55)	<0.001
**Dementia**				
Age	1.02 (0.98–1.06)	0.377	1.04 (0.99–1.09)	0.118
Sex (female)	1.21 (0.58–2.54)	0.609	2.59 (0.78–8.38)	0.116
Education, year	1.00 (0.94–1.07)	0.992	1.01 (0.92–1.10)	0.856
Family history of dementia	0.71 (0.34–1.50)	0.372	0.99 (0.44–2.23)	0.977
GA exposure	0.40 (0.18–0.87)	0.022	0.36 (0.15–0.84)	0.019
Ever smoking, *n* (%)	1.22 (0.54–2.81)	0.636	4.02 (1.13–14.13)	0.030
History of alcohol consumption, *n* (%)	0.69 (0.32–1.45)	0.323	0.54 (0.22–1.35)	0.184
Diabetes mellitus	1.34 (0.56–3.35)	0.521	1.98 (0.76–5.32)	0.169
Hypertension	0.52 (0.25–1.07)	0.081	0.52 (0.23–1.15)	0.109
Dyslipidemia	0.63 (0.30–1.33)	0.225	0.61 (0.27–1.36)	0.229
Comorbidity counts	0.83 (0.46–1.52)	0.537	1.09 (0.56–2.12)	0.808
APOE ε4 carrier	0.60 (0.29–1.23)	0.168	0.55 (0.25–1.17)	0.125
PET-amyloid+	1.35 (0.55–3.24)	0.510	1.82 (0.66–4.97)	0.244

Abbreviations: GDS, global deterioration scale; OR, odds ratio; CI, confidential interval; MCI, mild cognitive impairment; GA, general anesthesia; PET, positron emission tomography.

Table S4. Effect modification of the association between GA exposure and cognitive severity by APOE ε4 carrier status and amyloid positivity.

(a) Effect Modification by *APOE* ε4 Carrier Status						
**Diagnostic Group**	**Outcome**	**APOE ε4**	***n***	**OR (95% CI) [a]**	***p***	***p* for Interaction**
Normal	CDR-SB	Non-carrier	210	2.68 (1.28–5.56)	0.009	0.196
		Carrier	48	1.30 (0.29–5.86)	0.726	
	GDS	Non-carrier	210	2.71 (1.37–5.44)	0.004	0.319
		Carrier	48	2.09 (0.49–10.17)	0.321	
MCI	CDR-SB	Non-carrier	132	0.36 (0.09–1.13)	0.082	0.709
		Carrier	113	0.25 (0.07–0.73)	0.009	
	GDS	Non-carrier	132	0.17 (0.00–1.71)	0.155	0.992
		Carrier	113	0.52 (0.13–1.69)	0.290	
Dementia	CDR-SB	Non-carrier	88	2.82 (0.77–10.22)	0.114	0.017
		Carrier	97	0.22 (0.02–1.03)	0.055	
	GDS	Non-carrier	88	0.61 (0.20–1.78)	0.366	0.125
		Carrier	97	0.64 (0.22–1.70)	0.372	
**(b) Effect Modification by Amyloid Positivity**						
**Diagnostic Group**	**Outcome**	**Amyloid**	***n***	**OR (95% CI) [a]**	***p***	***p* for Interaction**
Normal	CDR-SB	Negative	213	3.24 (1.45–7.18)	0.004	0.079
		Positive	45	0.68 (0.18–2.55)	0.558	
	GDS	Negative	213	3.11 (1.55–6.32)	0.002	0.351
		Positive	45	0.74 (0.18–3.05)	0.672	
MCI	CDR-SB	Negative	116	0.56 (0.10–2.18)	0.426	0.350
		Positive	129	0.19 (0.06–0.51)	0.001	
	GDS	Negative	116	— [b]	—	0.993
		Positive	129	0.48 (0.12–1.47)	0.208	
Dementia	CDR-SB	Negative	35	— [b]	—	0.946
		Positive	150	0.67 (0.22–1.79)	0.439	
	GDS	Negative	35	0.78 (0.12–3.88)	0.772	0.349
		Positive	150	0.59 (0.27–1.26)	0.178	

CDR-SB, Clinical Dementia Rating Sum of Boxes; CI, confidence interval; GA, general anesthesia; GDS, Global Deterioration Scale; MCI, mild cognitive impairment; OR, odds ratio. [a] Odds ratios for the high- versus low-severity category associated with GA exposure were estimated within each stratum using Firth's penalized logistic regression, adjusted for age, sex, and years of education. The ***p*** for interaction was derived from a multiplicative interaction term (GA × modifier) in the multivariable logistic regression model adjusted for the full set of covariates. [b] Not estimated owing to sparse cells (fewer than three participants in the high-severity category). Interaction analyses were exploratory and should be interpreted cautiously because of the limited sample size in several subgroup strata.
